# Exact Power and Sample Size Calculations for the Two One-Sided Tests of Equivalence

**DOI:** 10.1371/journal.pone.0162093

**Published:** 2016-09-06

**Authors:** Gwowen Shieh

**Affiliations:** Department of Management Science, National Chiao Tung University, Hsinchu, 30010, Taiwan; University of New South Wales, AUSTRALIA

## Abstract

Equivalent testing has been strongly recommended for demonstrating the comparability of treatment effects in a wide variety of research fields including medical studies. Although the essential properties of the favorable two one-sided tests of equivalence have been addressed in the literature, the associated power and sample size calculations were illustrated mainly for selecting the most appropriate approximate method. Moreover, conventional power analysis does not consider the allocation restrictions and cost issues of different sample size choices. To extend the practical usefulness of the two one-sided tests procedure, this article describes exact approaches to sample size determinations under various allocation and cost considerations. Because the presented features are not generally available in common software packages, both R and SAS computer codes are presented to implement the suggested power and sample size computations for planning equivalence studies. The exact power function of the TOST procedure is employed to compute optimal sample sizes under four design schemes allowing for different allocation and cost concerns. The proposed power and sample size methodology should be useful for medical sciences to plan equivalence studies.

## Introduction

Equivalence tests have been widely adopted for demonstrating the bioequivalence between two drug formulations in biopharmaceutical studies. The notion of equivalence between treatment effects is equally relevant and potentially useful in other of research fields such as medical sciences. Although it is not the uniformly most powerful test and more powerful tests exist, the two one-sided tests (TOST) procedure proposed by Schuirmann [1] and Westlake [2] is the most common method for equivalence assessment under a two-group parallel design. A comprehensive review of the different types of equivalence tests was presented in Meyners [3]. Further details on the design and analysis of equivalence studies can be found in Chow and Liu [4], Chow, Shao, and Wang [5], Hauschke, Steinijans, and Pigeot [6], and Wellek [7].

It should be noted that the logic of the traditional difference-based tests and the formal equivalence-based tests are fundamentally distinct. Rogers et al. [8] emphasized that the traditional test and the equivalence test are not mutually exclusive. If both test procedures are performed, it is possible that both will be rejected, that neither will be rejected, or that one will be rejected and the other will not be rejected. Therefore, failing to reject a no-difference hypothesis test does not necessarily support the conclusion of equivalence as was stressed in Blackwelder [9]. Also, Cribbie, Gruman, and Arpin-Cribbie [10], Parkhurst [11], and Schuirmann [12] conducted comprehensive comparisons about the intrinsic appropriateness and theoretical properties between the TOST procedure and the two-sample *t* test for assessing the equivalence of two treatment means. More importantly, Allan and Cribbie [13] emphasized that the traditional tests are often inappropriately applied to establish equivalence in the psychological literature.

To improve the underutilized situation, the TOST procedure is strongly recommended, instead of the two-sample *t* test, when the research objective is to determine whether two treatment means are sufficiently near each other to be considered equivalent. The theoretical justification and computational ease are vital features of the TOST procedure for making statistical inferences. However, an empirical study requires adequate statistical power and sufficient sample size to detect designated hypotheses and examine research questions. The corresponding power calculations and sample size determinations must also be considered for a viable procedure to extend the applicability in planning research designs. Accordingly, considerable attention has been devoted to the power and sample size issues of the TOST procedure in the literature. Because the power function of the TOST is complicated in form, various expressions, approximations and computing algorithms have been proposed and discussed from different perspectives. The key findings are documented in Bristol [14], Chow, Shao, and Wang [15], Chow and Wang [16], Diletti, Hauschke, and Steinijans [17], Liu and Chow [18], Muller-Cohrs [19], Phillips [20], Schuirmann [12], Siqueira, Whitehead, Todd, and Lucini [21], and Wang and Chow [22], among others. It is essential to note that the inferential procedure and theoretical property of the TOST under a two-group parallel design immediately extend to the two-sequence and two-period crossover designs and the replicated crossover designs as explicated in Chow, Shao, and Wang [15], Chow and Wang [16], Siqueira et al. [21], and Wang and Chow [22].

Although the desirable properties of the two one-sided tests of equivalence, including the exact power function, have been well documented in the literature, the associated power and sample size calculations were illustrated mainly for selecting the most appropriate approximate method. At first sight, the approximate power functions are comparatively easy to use and seem to give practically useful results. But it does not retain all of the critical characteristics of the model configurations, and thus, there is no guarantee that the resulting sample size techniques will always give reliable performance. It was noted in Siqueira et al. [21] that simple approximations are satisfactory under certain conditions and the difficulty of calculating the sample size is not necessarily reduced by using the approximate formulas. On the other hand, with the advance of computer technology and the general availability of statistical software, computational simplicity is no longer a primary focus. Most importantly, the superiority of exact techniques in terms of accuracy is irreplaceable. Therefore, the exact power and sample size calculations should be considered instead. It is prudent to note that Bristol [14] and Schuirmann [12] described a particularly attractive and convenient expression for the exact power function of the TOST that can be readily implemented with the embedded normal and chi-square distribution functions in standard software systems.

Among others, Jan and Shieh [23] noted that conventional power analysis and sample size determination do not address matters of allocation restrictions and cost issues. However, researchers have been exploring design strategies that accommodate different constraints of the allocation structure and project funding while maintaining adequate power. Specifically, the allocation ratio of group sizes was fixed in the calculation of sample size for examining independent proportions in Fleiss, Tytun and Ury [24], while Heilbrun and McGee [25] considered sample size problem for the comparison of normal means when one sample size is specified in advance. Moreover, in an actual experiment, the available resources are generally limited and the cost for treating a subject often varies with treatment groups. For example, Nam [26] presented optimal sample sizes to maximize power for the comparison of the treatment and control under budget constraints. In contrast, Allison et al. [27] advocated designing statistically powerful studies while minimizing costs.

In view of the insufficient consideration on the exact sample size methodology in the literature, the present article aims to contribute to the development of optimal sample size determinations for the design of equivalence studies in two ways. First, the exact power function of the TOST procedure is employed to compute optimal sample sizes under four design schemes allowing for different allocation and cost concerns. The allocation schemes include (a) the ratio of group sizes is given, and (b) one sample size is specified. Moreover, the cost implications suggest optimally assigning subjects (a) to attain maximum power performance for a fixed cost, and (b) to meet a designated power level for the least cost. Second, because existing software packages do not accommodate power and sample size considerations with the same degree of generality as described in this article, computer algorithms are developed to facilitate the implementation of the suggested procedures. The proposed power and sample size methodology should be useful for medical sciences to plan equivalence studies.

## Methods

### Two one-sided tests procedure

Consider independent random samples from two normal populations with the following formulations:

$$X_{ij}∼N(\mu_{i},\sigma^{2}),$$ (1)

where μ_*i*_, σ^2^ are unknown parameters, *j* = 1, …, *N*_*i*_, and *i* = 1 and 2. For detecting the group effect μ_*d*_ = μ_1_ –μ_2_ in terms of the hypothesis H_0_: μ_*d*_ = 0 versus H_1_: μ_*d*_ ≠ 0, the common two-sample *t* statistic has the form

$$T=\frac{{\bar{X}}_{1}-{\bar{X}}_{2}}{S*},$$ (2)

where ${\bar{X}}_{1}=\sum_{j=1}^{N_{1}}X_{1j}/N_{1},\;{\bar{X}}_{2}=\sum_{j=1}^{N_{2}}X_{2j}/N_{2},\;S^{*}{}^{2}=S^{2}(1/N_{1}+1/N_{2}),\;S^{2}=\{(N_{1}-1)S_{1}^{2}+(N_{2}-1)S_{2}^{2}\}/\nu ,\;S_{1}^{2}=\sum_{j=1}^{N_{1}}{\left(X_{1j}-{\bar{X}}_{1}\right)}^{2}/(N_{1}-1),\;S_{2}^{2}=\sum_{j=1}^{N_{2}}{\left(X_{2j}-{\bar{X}}_{2}\right)}^{2}/(N_{2}-1)$, and ν = *N*_1_ + *N*_2_−2.

The primary focus of this article is the test of equivalence and without loss of generality, the null and alternative hypotheses are expressed as

$${\text{H}}_{0}:\mu_{d}\leq –\Delta {\;\text{or}\;\mu }_{d}\geq \Delta \;{\text{versus H}}_{1}:\;-\Delta <\mu_{d}<\Delta ,$$ (3)

where Δ (> 0) is a priori constant that represents the minimal difference for declaring equivalent means. It follows from the TOST procedure proposed by Schuirmann [1] and Westlake [2] that the null hypothesis is rejected at the significance level α if

$$T_{1}=\frac{{\bar{X}}_{1}-{\bar{X}}_{2}+\Delta }{S*}>t_{v,\alpha }\;\text{and}\;T_{2}=\frac{{\bar{X}}_{1}-{\bar{X}}_{2}-\Delta }{S*}<-t_{v,\alpha },$$ (4)

where *t*_ν, α_ is the upper 100·α-th percentile of the *t* distribution with degrees of freedom ν.

The exact power function Ψ_*E*_ of the TOST procedure is presented in Equation A2 of S1 File. The numerical computation of exact power requires the evaluation of the cumulative distribution function of a standard normal variable and the one-dimensional integration with respect to a chi-square probability distribution function. Since all related functions are presented in major statistical packages, the exact computations can be conducted with current computing systems. For advance planning of equivalence studies, the presented power function Ψ_*E*_ can be employed to calculate the sample sizes {*N*_1*E*_, *N*_2*E*_} needed to attain the specified power (1 –β) for the chosen significance level α, the model configurations {μ_*d*_, σ^2^}, and the equivalence threshold Δ. In order to enhance the applicability of the TOST procedure, optimal sample size algorithms are described in the subsequent section for four design schemes under different allocation and cost considerations. The R [28] and SAS/IML [29] programs employed to perform the corresponding sample size calculations are available in S3 File and S4 File, respectively. While the optimal power and sample size considerations are primarily illustrated for the equivalence evaluations of two-group parallel designs, they may be directly extended to equivalence problems of two-sequence and two-period crossover designs as explicated in S2 File.

For illustration, simulation study was conducted to demonstrate the suggested exact approach for power and sample size calculations. The empirical assessment examines the two mean difference patterns and six standard deviation values presented in Table V of Siqueira et al. [21]. Specifically, the two sets of mean differences and standard deviations are μ_*d*_ = {0.0, 0.1} and σ = {0.10, 0.12, 0.14, 0.16, 0.18, 0.20}, respectively. Also, the chosen sample sizes were determined to achieve the power level 0.80 with the gold standard method reported in Siqueira et al. [21]. They only considered balanced design with the sample sizes *N*_1_ = *N*_2_ = *N* and the gold standard method is an approximation with the power function Ψ_*A*_ given in Equation A5 of S1 File. Accordingly, the exact power function Ψ_*E*_ presented in Equation A3 is employed to compute the attained power for the twelve model configurations with α = 0.05 and Δ = 0.2231.

Moreover, estimates of the true power associated with given sample size and parameter configuration were computed via Monte Carlo simulation of 100,000 independent data sets. For each replicate, (*N*_1_, *N*_2_) normal outcomes are generated with the two-sample parallel design. Then, the test statistics *T*_1_ and *T*_2_ are computed and the simulated power is the proportion of the 100,000 replicates with the test statistics *T*_1_ > *t*_ν, 0.05_ and *T*_2_ < −*t*_ν, 0.05_. The adequacy for power and sample size calculation is determined by the difference between the simulated power and computed power. The computed power, simulated power, and the corresponding difference are summarized in Table 1 for the examined model settings. An inspection of the summarized results reveals that the suggested exact method based on the power function Ψ_*E*_ produces almost identical results with the simulation for all twelve cases. Specifically, the resulting absolute differences are all less than 0.003 and the largest discrepancy 0.0025 is incurred by the situation with μ_*d*_ = 0.1, σ = 0.12, and *N* = 13. Hence, the presented exact approach and computer algorithm have the distinct advantage in computational accuracy.

**Table 1:** The computed power and simulated power of the two one-sided test for α = 0.05, Δ = 0.2231, and equal sample sizes *N*_1_ = *N*_2_ = *N*.

μ_*d*_	σ	*N*	Computed power	Simulated power	Difference
0.00	0.10	5	0.8823	0.8806	0.0017
	0.12	6	0.8220	0.8218	0.0002
	0.14	8	0.8333	0.8331	0.0002
	0.16	10	0.8238	0.8242	–0.0004
	0.18	12	0.8049	0.8035	0.0014
	0.20	15	0.8181	0.8192	–0.0011
0.10	0.10	9	0.8033	0.8050	–0.0017
	0.12	13	0.8148	0.8123	0.0025
	0.14	17	0.8062	0.8065	–0.0003
	0.16	22	0.8066	0.8068	–0.0002
	0.18	28	0.8110	0.8106	0.0004
	0.20	34	0.8070	0.8083	–0.0013

### Design schemes

With the exact power function of the TOST procedure, this study examines research designs with the allocating and budgetary constraints. First, the ratio *r* = *N*_2_/*N*_1_ between the two group sizes may be fixed in advance, so the task is to decide the minimum sample size *N*_1_ (*N*_2_ = *rN*_1_) required to achieve the specified power level. Second, one of the two sample sizes, say *N*_2_, may be pre-assigned, and so the smallest size *N*_1_ required to satisfy the designated power should be found. Third, what is the least cost for a research study to maintain its desired power level? Fourth, how can the maximum power be attained in a scientific investigation with a limited budget?

#### Design I: sample size ratio is fixed

Consider the scenario that the sample size ratio *r* = *N*_2_/*N*_1_ is pre-assigned, and for ease of illustration, the ratio is assumed as *r* ≥ 1. The common balanced design with equal sample sizes is the special case with *r* = 1. An incremental process can be conducted to determine the minimum sample size *N*_1_ needed to attain the specified power 1 –β for the chosen significance level α and parameter values {μ_*d*_, σ^2^, Δ}. For comparative purpose and computational ease, the approximate normal distribution *T* ⩪ *N*(λ, 1) provides a convenient solution where 0λ = μ_*d*_/σ* and σ*^2^ = σ^2^(1/*N*_1_ + 1/*N*_2_). To simplify the computation, the starting sample size *N*_1*Z*_ computed by the normal approximation would be the smallest integer that satisfies the inequality *N*_1*Z*_ ≥ (1 + /*r*)σ^2^(*z*_α_ + *z*_β_)^2^/(Δ–|μ_*d*_|)^2^ where *z*_α_ and *z*_β_ are the upper 100·αth and 100·βth percentiles of the standard normal distribution, respectively.

#### Design II: one sample size is fixed

Without loss of generality, the sample size *N*_2_ of the second group is held constant. Just as in the previous case, the minimum sample size *N*_1_ needed to ensure the specified power 1 –β can be found by an iterative search for the chosen significance level α and parameter values {μ_*d*_, σ^2^, Δ}. The starting sample size *N*_1*Z*_, based on the normal approximation as is described in the previous situation, is chosen as the smallest integer that satisfies the inequality *N*_1*Z*_ ≥ 1/{(Δ–|μ_*d*_|)^2^/[σ^2^(*z*_α_ + *z*_β_)^2^]–/*N*_2_}.

#### Design III: total cost is fixed and the actual power needs to be maximized

Suppose *C*_*F*_ is the overhead cost of the study, and *C*_1_ and *C*_2_ are the costs per subject in the first and second groups, respectively; then the total cost of the study is *C* = *C*_*F*_ + *C*_1_*N*_1_ + *C*_2_*N*_2_. Accordingly, the traditional consideration of the total number of subjects can be viewed as a special case of the cost function *C*, with *C*_*F*_ = 0 and *C*_1_ = *C*_2_ = 1. Within the normality context, Pentico [30] showed that the optimal allocation with different unit sampling costs is when the ratio of the sample sizes assumes the equality *N*_1_/*N*_2_ = $C_{2}^{1/2}/C_{1}^{1/2}$. For a fixed value of total cost *C* and a specified fixed cost *C*_*F*_, the maximum power is obtained with the sample size combination

$$N_{1Z}=\frac{C_{2}^{1/2}(C-C_{F})}{C_{1}C_{2}^{1/2}+C_{2}C_{1}^{1/2}}\;\text{and}\;N_{2Z}=\frac{C_{1}^{1/2}(C-C_{F})}{C_{1}C_{2}^{1/2}+C_{2}C_{1}^{1/2}}.$$

Notably, the optimal property is valid only when the statistic *T* has a normal distribution. However, this is not the case here and a two-step procedure is conducted to find the exact optimum. First, a detailed power assessment is performed for the sample size combinations {*N*_1_, *N*_2_} with *N*_1_ from *N*_1*min*_ to *N*_1*max*_ and *N*_2_ = *Floor*[(*C*–*C*_*F*_−*C*_1_*N*_1_)/*C*_2_], where *N*_1*min*_ = *Floor*(*N*_1*Z*_)– 3, *N*_1*max*_ = *Floor*[{*C*–*C*_*F*_−*C*_2_(*Floor*(*N*_2*Z*_)– 3)}/*C*_1_], and the function *Floor*(*a*) returns the largest integer that is less than or equal to *a*. Second, the optimal sample size allocation is the one giving the largest power.

#### Design IV: target power is fixed and the total cost needs to be minimized

In addition to the preceding scenario with limited budget, a distinct approach to take into account both power and cost issues is to find the optimal sample size combination which minimizes the total cost and attains the pre-chosen target power. In view of the discrete character of sample size, the exact procedure is conducted in three steps.

First, in order to achieve the nominal power 1 –β while minimizing total cost *C* = *C*_*F*_ + *C*_1_*N*_1*Z*_ + *C*_2_*N*_2*Z*_, a nearly optimal sample size combination under the normal distribution for *T* is

$$N_{1Z}=\frac{(1+C_{2}^{1/2}/C_{1}^{1/2})\sigma^{2}{\left(z_{\alpha }+z_{\mathrm{\beta *}}\right)}^{2}}{{\left(\Delta -\left|\mu_{d}\right|\right)}^{2}}\;\text{and}\;N_{2Z*}=\frac{(1+C_{1}^{1/2}/C_{2}^{1/2})\sigma^{2}{\left(z_{\alpha }+z_{\mathrm{\beta *}}\right)}^{2}}{{\left(\Delta -\left|\mu_{d}\right|\right)}^{2}}$$

where *z*_β*_ = *z*_β/2_ if μ_*d*_ = 0, and *z*_β*_ = *z*_β_ if μ_*d*_ ≠ 0. It can be seen that *N*_1Z*_/*N*_2Z*_ = $C_{2}^{1/2}/C_{1}^{1/2}$ and σ^2^(1/*N*_1Z*_ + 1/*N*_2Z*_) = (σ–|μ_*d*_|)^2^/(*z*_α_ + *z*_β*_)^2^. Then, the power computation and cost evaluation are conducted for sample size combinations with *N*_1_ from *N*_1*min*_ to *N*_1*max*_ and a proper value of *N*_2_ ≥ *Floor*[1/{(Δ–|μ_*d*_|)^2^/[σ^2^(*z*_α_ + *z*_β*_)^2^] – 1/*N*_1_}] satisfying the required power, where *N*_1*min*_ = *max*{5, *Ceil*(*N*_1Z*_)– 2}, *N*_1*max*_ = *Ceil*(*N*_1Z*_) + 10, the *max* function selects the largest value of the elements, and the function *Ceil*(*a*) returns the smallest integer that is greater than or equal to *a*. Second, the optimal sample size allocation is the one giving the smallest cost while maintaining the specified power level. Third, there may be more than one combination giving the same amount of least cost. A further screening and selection process is conducted to find the one {*N*_1*E*_, *N*_2*E*_} producing the largest power.

## Results

To illustrate the computational aspects of the suggested procedures for design planning, the example of Minnesota Multiphasic Personality Inventory (MMPI) similarities between alcohol and drug-dependent subjects presented in Rogers et al. [8] is extended here to sample size determinations for equivalence testing under various design schemes. Detailed discussions and related results of the MMPI differences between alcoholics and drug abusers can be found in Cannon, Bell, and Fowler [31].

Due to the prospective nature of advance research planning, the general guidelines suggest that typical sources like published finding or expert opinion can offer plausible and reasonable planning values for the vital characteristics of mean effects, variance components, and equivalence threshold. As an illustration of sample size determination for planning equivalence study, the reported summary statistics of the Masculinity-Femininity scale for the drug and alcohol-dependent groups are modified as the population means and variance. Specifically, μ_*d*_ = 61.4–59.2 = 2.2, σ = 9.78. With these specifications, significance level α = 0.05, and equivalence bound Δ = 5.92 (10% of the MMPI scores of the alcoholic subjects), the numerical computation showed that the resulting power for the TOST is Ψ_*E*_ = 0.7711 for the reported sample sizes {*N*_1_, *N*_2_} = {49, 207} of the MMPI study. The attained power is slightly less than the fairly common and somehow minimal level of 0.80.

In order to warrant a decent chance of assessing the equivalence property with the pre-assigned the sample size ratio *r* = *N*_2_/*N*_1_ = 4, the optimal sample sizes {54, 216} are required to attain the designated power 0.80. Alternatively, when the sample size *N*_2_ = 210 is fixed beforehand, it requires a group size of *N*_1_ = 55 in order to achieve the selected power 0.80. However, it is important to take budget issues into account. For illustration, assume the unit sampling costs for the two treatment groups are *C*_1_ = 4 and *C*_2_ = 1. Under cost consideration with the overhead cost *C*_*F*_ = 0 and the total budget *C* = 400 units, the optimal sample size solution is {67, 132} which has an actual power of 0.8111. On the other hand, the optimal sample sizes {65, 128} are required to attain the designated power 0.80 with the least total cost. The detailed computation showed that the attained power and total cost are 0.8005 and 388, respectively. The prescribed parameter configurations are incorporated in the user specifications of the supplementary R and SAS/IML programs. Researchers can easily identify the statements containing the exemplifying values in the computer code and then modify the programs to accommodate their own model specifications. Nonetheless, the suggested procedures will yield accurate power calculations and sample size determinations provided that all the required information is properly specified.

## Conclusions

Many studies are designed explicitly to show that two treatments are functionally equivalent or that a new method is as effective as a well-established method under the same condition. Under such circumstance, the traditional tests are inappropriate to establish equivalence, because failing to reject a no-difference hypothesis test does not necessarily support the conclusion of equivalence. Notably, the TOST procedure for establishing statistical equivalence has been used effectively across a wide range of research disciplines. As a contrasting and concrete example, the TOST procedure were illustrated with the MMPI equivalence evaluations between alcohol and drug-dependent subjects in Rogers et al. [8], while the previous study of Cannon, Bell, and Fowler [31] focused on the research issues of MMPI differences between alcoholics and drug abusers. Consequently, it is advisable that investigators should determine when equivalency testing is useful and delineate meaningful equivalence bounds relative to the substantive issues in their expert areas of research.

To enhance the usefulness of TOST methodology, it is prudent to develop a full account of computer programs for implementing the necessary calculations in equivalence studies. Evidently, the lack of efficient and convenient computer software impedes the practical use of equivalence tests and the theoretical development of equivalence research. This article examines the power and sample size problem of equivalence testing for the means from two independent and normally distributed populations with an unknown variance. The exact power function of the TOST procedure is described and employed to compute the optimal sample sizes under various allocation and cost considerations. In view of the importance of power and sample size calculations in design planning and the limited features of available software packages, computer programs are developed to facilitate the usage of the proposed techniques. Overall, the illustrated power and sample size calculations and accompanying algorithms reinforce the theoretical and practical implications of TOST in equivalence studies.

## Supporting Information

S1 File Power function of the two one-sided tests. (DOCX)

S2 File The two one-sided tests for 2 × 2 crossover designs. (DOCX)

S3 File R programs. (DOCX)

S4 File SAS programs. (DOCX)
