# Supplementary material for: Exact Power and Sample Size Calculations for the Two One-Sided Tests of Equivalence
Source: PLoS One. 2016 Sep 6;11(9):e0162093. doi: 10.1371/journal.pone.0162093 (PMC5012670; doi:10.1371/journal.pone.0162093)
Supplement: S1 File — (DOCX) [file pone.0162093.s001.docx]

**S1 File**

Power function of the two one-sided tests

In general, the statistic *T* defined in Equation 2 has a noncentral *t* distribution

*T* ~ *t*(ν, λ), (A1)

where *t*(ν, λ) is the noncentral *t* distribution with degrees of freedom ν, and noncentrality parameter λ =μ*_d_*/σ* with σ*^2^ = σ^2^(1/*N*_1_ + 1/*N*_2_). Accordingly, the power function of the TOST procedure given in Equation 4 is denoted by

Ψ*_E_* = *P*{*T*_1_ > *t* and *T*_2_ < –*t*} = *P*{ –Δ + *tS** *<* _1_ – _2_ < Δ – *tS**}. (A2)

Note that the inequality requires the upper bound is greater than the lower bound in the probability evaluation of _1_ – _2_. Hence, the power function is valid (Ψ*_E_* > 0) only if *S** < Δ/*t*. Consequently, the rejection region of the TOST procedure has an isosceles triangular shape as illustrated in Meyners [3] (Figure 5a) and Schuirmann [12] (Figure 2).

For the purpose of analytic clarification and numerical assessment, an alternative and elaborated formulation of Ψ*_E_* is presented next. It follows from the fundamental assumption that *Z* = (_1_ – _2_ – μ*_d_*)/σ* ~ *N*(, 1), *V* = ν*S*^2^/σ^2^ ~ χ^2^(ν), χ^2^(ν) denotes the chi-square distribution with ν degrees of freedom, and *Z* and *V* are independent. Accordingly, the power function Ψ*_E_* can be rewritten as

Ψ*_E_* = *E_V_*[Ψ(*U*) – Ψ(*L*)], (A3)

where *U* = (Δ – μ*_d_*)/σ* – *t*(*V**/ν), *L* = –(Δ + μ*_d_*)/σ* + *t*(*V**/ν), *V** = *min*{*V*, (ν⋅Δ^2^)/(σ*^2^*t*)}, Ψ(⋅) is the cumulative density function of the standard normal distribution, and the expectation *E_V_* is taken with respect to the distribution *V*. It should be noted that the particular expression in Equation A3 is similar to those presented in Bristol [14] and Schuirmann [12]. Moreover, it is more analytically transparent than the formulation based on the bivariate noncentral *t* distribution considered in Diletti, Hauschke, and Steinijans [17], Phillips [20], and Wang and Chow [22].

It follows from extensive numerical examinations in Siqueira et al. [21] and Wang and Chow [22] that *P*{*S** ≥ Δ/*t*} 0 across a wide range of model configurations. This vital recognition permits computational simplification of the exact power. Specifically, the power function Ψ*_E_* can be approximated by

Ψ*_E_* = *P*{_1_ – _2_ < Δ – *tS**, Δ > *tS**} – *P*{_1_ – _2_ < –Δ + *tS**, Δ > *tS**}

*P*{_1_ – _2_ < Δ – *tS**} – *P*{_1_ – _2_ < –Δ + *tS**}

= *P*{*T*_2_ < –*t*} – *P*{*T*_1_ < *t*},

where *T*_1_ ~ *t*(ν, Δ_1_), Δ_1_ = (μ*_d_* + Δ)/σ*, *T*_2_ ~ *t*(ν, Δ_2_), and Δ_2_ = (μ*_d_* – Δ)/σ*. Hence, a useful approximate power of the TOST is

Ψ*_A_* = *P*{*t*(ν, Δ_2_) < –*t*} – *P*{*t*(ν, Δ_1_) < *t*}. (A4)

It is interesting to see that similar expressions have been presented in Siqueira et al. [21] and Wang and Chow [22] from different perspectives.
