# Supplementary material for: Exact Power and Sample Size Calculations for the Two One-Sided Tests of Equivalence
Source: PLoS One. 2016 Sep 6;11(9):e0162093. doi: 10.1371/journal.pone.0162093 (PMC5012670; doi:10.1371/journal.pone.0162093)
Supplement: S2 File — (DOCX) [file pone.0162093.s002.docx]

**S2 File**

The two one-sided tests for 2 × 2 crossover designs

Consider the standard two-sequence and two-period crossover design

*Y_ijk_* = μ + *F_ij_* + *P_j_* + *S_ik_* + ε*_ijk_* (B1)

where *Y_ijk_* is the outcome for the *k*th subject in the *i*th sequence and *j*th period, μ is the grand mean, *F_ij_* is the formulation effect, *P_j_* is the fixed period effect, *S_ik_* is the random subject effect, and ε*_ijk_* is the random error for *i* = 1 and 2, *j* = 1 and 2, and *k* = 1, …, *N_i_*. Under the standard setting, the formulation effects are expressed as *F*_11_ = *F*_22_ = μ*_R_* and *F*_11_ = *F*_22_ = μ*_T_* for the reference product and test product, respectively, {*S_ik_*} are independent *N*(0, σ) variables, and {ε*_ijk_*} are independent *N*(0, σ) variables with σ = σ = σ and σ = σ = σ. Also, it is assumed that *P*_1_ + *P*_2_ = μ*_R_* + μ*_T_* = 0. For the inference of the difference between the test product and reference product μ*_D_* = μ*_T_* – μ*_R_*, it is useful to consider the mean difference – where = *D_ik_*/*N_i_*, *D_ik_* = (*Y_i_*_2_*_k_* – *Y_i_*_1_*_k_*)/2 for *i* = 1 and 2, and *k* = 1, …, *N_i_*. With the prescribed model assumptions, the mean difference – has the distribution

– ~ *N*(μ*_D_*, σ),

where σ = σ(1/*N*_1_ + 1/*N*_2_) and σ = *Var*(*D_ik_*) = (σ + σ)/4. Moreover, *S* = (*D_ik_* – )^2^/ν is an unbiased estimator of σ and (ν*S*)/σ has a chi-square distribution with degrees of freedom ν = *N*_1_ + *N*_2_ – 2. Hence, a *t* statistic can be constructed as

*T_D_* = , (B2)

where *S* = *S*(1/*N*_1_ + 1/*N*_2_). It is noteworthy that the statistic *T_D_* has a similar formulation as the *T* statistic given in Equation 2. Thus, the theoretical property and inference procedure for the TOST of equivalence under a two-group parallel design readily apply to the TOST of equivalence for a two-sequence and two-period crossover design. Specifically, the test of equivalence in terms of the null and alternative hypotheses

H_0_: μ*_D_* ≤ –Δ or μ*_D_* ≥ Δ versus H_1_: –Δ < μ*_D_* < Δ (B3)

can be conducted by rejecting the null hypothesis at the significance level α if

*T_D_*_1_ = > *t* and *T_D_*_2_ = < –*t*, (B4)

where *t* is the upper 100·α-th percentile of the *t* distribution with degrees of freedom ν. Also, the statistic *T_D_* has a noncentral *t* distribution with degrees of freedom ν and noncentrality parameter λ*_D_* =μ*_D_*/σ:

*T_D_* ~ *t*(ν, λ*_D_*). (B5)

An argument similar to that for the proof of the power function Ψ*_E_* defined in Equation A2, the corresponding power function of the TOST procedure given in Equation B4 is

Ψ*_DE_* = *P*{*T_D_*_1_ > *t* and *T_D_*_2_ < –*t*} = *P*{ –Δ + *tS* *<* _1_ – _2_ < Δ – *tS*}. (B6)

It is of methodological importance to stress the close resemblance between the TOST and associated follow-up procedures under the two frameworks of the two-group parallel design and the 2 × 2 crossover design. Accordingly, the approximate sample size methods for the 2 × 2 crossover designs described in Chow and Wang [16] and Siqueira et al. [21] can immediately be improved with the exact approach. On the other hand, the exact power function given in Shen, Russek-Cohen, and Slud [32] for the two-sequence and two-period crossover design can be viewed as a direct application of the exact power function given in Bristol [14] and Schuirmann [12] under the standard two-group scenario. However, this vital connection and informative phenomenon was not recognized in Shen, Russek-Cohen, and Slud [32]. Also, the monotonicity property of a *t* distribution derived in Shen, Russek-Cohen, and Slud [32] was already documented in Corollary 4.3 by Ghosh [33]. It should be emphasized that the two R computer programs for power and sample size calculations in Shen, Russek-Cohen, and Slud [32] are confined to the balanced case with *N*_1_ = *N*_2_. In contrast, the developed R and SAS computer codes (A1, A2, B1, and B2) are applicable for both balanced and unbalanced structures. More importantly, the additional computer algorithms (A3-A5 and B3-B5) provide more and versatile features for exact power analysis and sample size determination under different allocation and cost considerations that were not considered in Shen, Russek-Cohen, and Slud [32]. For pedagogic purposes, it is essential to note that the exact power and sample size procedures described here can also be extended to the TOST of equivalence for the more general setting of replicated crossover designs considered in Chow, Shao, and Wang [15] and Wang and Chow [22].

**References**

32. Shen M, Russek‐Cohen E, Slud EV. Exact calculation of power and sample size in bioequivalence studies using two one‐sided tests. Pharmaceutical Statistics. 2015; 14: 95-101.

33. Ghosh BK. Some monotonicity theorems for χ^2^, F and t distributions with applications. Journal of the Royal Statistical Society Series B. 1973; 35: 480-492.
