# Supplementary material for: Exact Power and Sample Size Calculations for the Two One-Sided Tests of Equivalence
Source: PLoS One. 2016 Sep 6;11(9):e0162093. doi: 10.1371/journal.pone.0162093 (PMC5012670; doi:10.1371/journal.pone.0162093)
Supplement: S3 File — (DOCX) [file pone.0162093.s003.docx]

S3 File-R programs

Program A1

R program for calculating the achieved power of the TOST procedure

function () {

#USER SPECIFICATIONS PORTION

n1<-49 #sample sizes

n2<-207

alpha<-0.05 #type I error rate

del<-5.92 #equivalence bound

mud<-2.2 #mean difference

sigma<-9.78 #standard deviation

#END OF SPECIFICATION

sigsq<-sigma^2

numint<-1000

lc<-numint+1

cl<-1e-10

coevecc<-c(1,rep(c(4,2),numint/2-1),4,1)

df<-n1+n2-2

tcrit<-qt(1-alpha,df)

nfac<-1/n1+1/n2

var<-sigsq*nfac

std<-sqrt(var)

cu<-(df*del^2)/(var*tcrit^2)

int<-cu-cl

intl<-int/numint

cvec<-cl+intl*(0:numint)

wcpdf<-(intl/3)*coevecc*dchisq(cvec,df)

st<-sqrt(cvec/df)*tcrit

epower<-sum(wcpdf*(pnorm((del-mud)/std-st)-pnorm((-del-mud)/std+st)))

print("alpha, del, mud, sigma, sigsq")

print(c(alpha,del,mud,sigma,sigsq),digits=4)

print("n1, n2, epower")

print(c(n1,n2,epower),digits=4)

}

Program A2

R program for calculating the optimal sample sizes {*N*_1_, *N*_2_} when the sample size ratio is fixed

function () {

#USER SPECIFICATIONS PORTION

nr<-4 #sample size ratio

alpha<-0.05 #type I error rate

power<-0.80 #nominal power

del<-5.92 #equivalence bound

mud<-2.2 #mean difference

sigma<-9.78 #standard deviation

#END OF SPECIFICATION

n1<-4

sigsq<-sigma^2

numint<-1000

lc<-numint+1

cl<-1e-10

coevecc<-c(1,rep(c(4,2),numint/2-1),4,1)

epower<-0

while (epower<power & n1<1001){

n1<-n1+1

n2<-nr*n1

df<-n1+n2-2

tcrit<-qt(1-alpha,df)

nfac<-1/n1+1/n2

var<-sigsq*nfac

std<-sqrt(var)

cu<-(df*del^2)/(var*tcrit^2)

int<-cu-cl

intl<-int/numint

cvec<-cl+intl*(0:numint)

wcpdf<-(intl/3)*coevecc*dchisq(cvec,df)

st<-sqrt(cvec/df)*tcrit

epower<-sum(wcpdf*(pnorm((del-mud)/std-st)-pnorm((-del-mud)/std+st)))

}

print("alpha, del, mud, sigma, sigsq, nr, power")

print(c(alpha,del,mud,sigma,sigsq,nr,power),digits=4)

print("n1, n2, epower")

print(c(n1,n2,epower),digits=4)

}

Program A3

R program for calculating the optimal sample size *N*_2_ when the sample size *N*_2_ is fixed

function () {

#USER SPECIFICATIONS PORTION

n2<-210 #sample size n2

alpha<-0.05 #type I error rate

power<-0.80 #nominal power

del<-5.92 #equivalence bound

mud<-2.2 #mean difference

sigma<-9.78 #standard deviation

#END OF SPECIFICATION

sigsq<-sigma^2

numint<-1000

lc<-numint+1

cl<-1e-10

coevecc<-c(1,rep(c(4,2),numint/2-1),4,1)

n1<-3

epower<-0

while (epower<power & n1<1001){

n1<-n1+1

df<-n1+n2-2

tcrit<-qt(1-alpha,df)

nfac<-1/n1+1/n2

var<-sigsq*nfac

std<-sqrt(var)

cu<-(df*del^2)/(var*tcrit^2)

int<-cu-cl

intl<-int/numint

cvec<-cl+intl*(0:numint)

wcpdf<-(intl/3)*coevecc*dchisq(cvec,df)

st<-sqrt(cvec/df)*tcrit

epower<-sum(wcpdf*(pnorm((del-mud)/std-st)-pnorm((-del-mud)/std+st)))

}

print("alpha, del, mud, sigma, sigsq, n2, power")

print(c(alpha,del,mud,sigma,sigsq,n2,power),digits=4)

print("n1, n2, epower")

print(c(n1,n2,epower),digits=4)

}

Program A4

R program for calculating the optimal sample sizes {*N*_1_, *N*_2_} to attain maximum power performance for a fixed cost

function () {

#USER SPECIFICATIONS PORTION

cf<-0 #cost coefficients

c1<-4

c2<-1

tcost<-400 #total cost

alpha<-0.05 #type I error rate

del<-5.92 #equivalence bound

mud<-2.2 #mean difference

sigma<-9.78 #standard deviation

#END OF SPECIFICATION

sigsq<-sigma^2

numint<-1000

lc<-numint+1

cl<-1e-10

coevecc<-c(1,rep(c(4,2),numint/2-1),4,1)

tc<-tcost-cf

n1z<-floor(tc*sqrt(c2)/(c1*sqrt(c2)+c2*sqrt(c1)))

n1min<-n1z-3

n2z<-floor(tc*sqrt(c1)/(c1*sqrt(c2)+c2*sqrt(c1)))

n2min<-n2z-3

n1max=floor((tc-c2*n2min)/c1)

ln<-n1max-n1min+1

n1vec<-seq(n1min,n1max)

n2vec<-floor((tc-c1*n1vec)/c2)

tcvec<-cf+c1*n1vec+c2*n2vec

powervec<-rep(0,ln)

for (j in seq(ln)) {

n1<-n1vec[j]

n2<-n2vec[j]

df<-n1+n2-2

tcrit<-qt(1-alpha,df)

nfac<-1/n1+1/n2

var<-sigsq*nfac

std<-sqrt(var)

cu<-(df*del^2)/(var*tcrit^2)

int<-cu-cl

intl<-int/numint

cvec<-cl+intl*(0:numint)

wcpdf<-(intl/3)*coevecc*dchisq(cvec,df)

st<-sqrt(cvec/df)*tcrit

epower<-sum(wcpdf*(pnorm((del-mud)/std-st)-pnorm((-del-mud)/std+st)))

powervec[j]<-epower

}

maxi<-seq(ln)[powervec==max(powervec)]

maxn1<-n1vec[maxi]

maxn2<-n2vec[maxi]

tcmax<-tcvec[maxi]

pmax<-powervec[maxi]

print("alpha, del, mud, sigma, sigsq")

print(c(alpha,del,mud,sigma,sigsq),digits=4)

print("cf, c1, c2, tcost")

print(c(cf,c1,c2,tcost),digits=4)

print("n1, n2, tc, epower")

print(c(maxn1,maxn2,tcmax,pmax),digits=4)

}

Program A5

R program for calculating the optimal sample sizes {*N*_1_, *N*_2_} to meet a designated power level for the least cost

function () {

#USER SPECIFICATIONS PORTION

cf<-0 #cost coefficients

c1<-4

c2<-1

alpha<-0.05 #type I error rate

power<-0.80 #nominal power

del<-5.92 #equivalence bound

mud<-2.2 #mean difference

sigma<-9.78 #standard deviation

#END OF SPECIFICATION

sigsq<-sigma^2

numint<-1000

lc<-numint+1

cl<-1e-10

coevecc<-c(1,rep(c(4,2),numint/2-1),4,1)

nrz<-sqrt(c1/c2)

za<-qnorm(1-alpha)

zb1<-qnorm(power)

zb2<-qnorm(1-(1-power)/2)

if (mud==0) zb<-zb2

else zb<-zb1

k<-(sigma*(za+zb)/(del-abs(mud)))^2

n1z<-ceiling((1+1/nrz)*k)

n2z<-ceiling(n1z*nrz)

n1min<-max(5,n1z-2)

n1max<-n1z+10

n1vec<-seq(n1min,n1max)

ln<-n1max-n1min+1

n2vec<-rep(0,ln)

powervec<-rep(0,ln)

for (j in seq(ln)) {

n1<-n1vec[j]

n2<-floor(1/(1/k-1/n1))

n2<-max(n2,6)

loop<-0

epower<-0

while (epower<power & loop<201){

n2<-n2+1

loop<-loop+1

df<-n1+n2-2

tcrit<-qt(1-alpha,df)

nfac<-1/n1+1/n2

var<-sigsq*nfac

std<-sqrt(var)

cu<-(df*del^2)/(var*tcrit^2)

int<-cu-cl

intl<-int/numint

cvec<-cl+intl*(0:numint)

wcpdf<-(intl/3)*coevecc*dchisq(cvec,df)

st<-sqrt(cvec/df)*tcrit

epower<-sum(wcpdf*(pnorm((del-mud)/std-st)-pnorm((-del-mud)/std+st)))

}

n2vec[j]<-n2

powervec[j]<-epower

}

tcvec<-cf+c1*n1vec+c2*n2vec

tcmin<-min(tcvec)

minivec<-seq(ln)[tcvec==tcmin]

n1minvec<-n1vec[minivec]

n2minvec<-n2vec[minivec]

pminvec<-powervec[minivec]

pmaxmin<-max(pminvec)

maxmini<-seq(length(pminvec))[pminvec==pmaxmin]

n1maxmin<-n1minvec[maxmini]

n2maxmin<-n2minvec[maxmini]

pmaxmin<-pminvec[maxmini]

print("alpha, power, del, mud, sigma, sigsq")

print(c(alpha,power,del,mud,sigma,sigsq),digits=4)

print("cf, c1, c2")

print(c(cf,c1,c2),digits=4)

print("n1, n2, epower, tc")

print(c(n1maxmin,n2maxmin,pmaxmin,tcmin),digits=4)

}
