# Supplementary material for: Exact Power and Sample Size Calculations for the Two One-Sided Tests of Equivalence
Source: PLoS One. 2016 Sep 6;11(9):e0162093. doi: 10.1371/journal.pone.0162093 (PMC5012670; doi:10.1371/journal.pone.0162093)
Supplement: S4 File — (DOCX) [file pone.0162093.s004.docx]

S4 File-SAS programs

Program B1

SAS IML program for calculating the achieved power of the TOST procedure

PROC IML;

*USER SPECIFICATION PORTION;

*SAMPLE SIZES;N1=49;N2=207;

*TYPE I ERROR;ALPHA=0.05;

*EQUIVALENCE BOUND;DEL=5.92;

*MEAN DIFFERENCE;MUD=2.2;

*STANDARD DEVIATIONS;SIGMA=9.78;

*END OF SPECIFICATION PORTION;

SIGSQ=SIGMA##2;NUMINT=1000;LC=NUMINT+1;CL=1E-10;

COEVECC=({1}||REPEAT({4 2},1,NUMINT/2-1)||{4 1})`;

DF=N1+N2-2;

TCRIT=TINV(1-ALPHA,DF);

NFAC=1/N1+1/N2;

VAR=SIGSQ#NFAC;STD=SQRT(VAR);

CU=(DF#DEL##2)/(VAR#(TCRIT##2));

INT=CU-CL;INTL=INT/NUMINT;

CVEC=CL+(INTL#(0:NUMINT))`;

WCPDF=(INTL/3)#COEVECC#PDF('CHISQ',CVEC,DF);

ST=SQRT(CVEC/DF)#TCRIT;

EPOWER=WCPDF`*(CDF('NORMAL',(DEL-MUD)/STD-ST)-CDF('NORMAL',(-DEL-MUD)/STD+ST));

PRINT ALPHA DEL MUD SIGMA SIGSQ;

PRINT N1 N2 EPOWER[FORMAT=8.4];

QUIT;

Program B2

SAS IML program for calculating the optimal sample sizes {*N*_1_, *N*_2_} when the sample size ratio is fixed

PROC IML;

*USER SPECIFICATION PORTION;

*SAMPLE SIZE RATIO;NR=4;

*TYPE I ERROR & NOMINAL POWER;ALPHA=0.05;POWER=0.8;

*EQUIVALENCE BOUND;DEL=5.92;

*MEAN DIFFERENCE;MUD=2.2;

*STANDARD DEVIATIONS;SIGMA=9.78;

*END OF SPECIFICATION PORTION;

N1=4;SIGSQ=SIGMA##2;NUMINT=1000;LC=NUMINT+1;CL=1E-10;

COEVECC=({1}||REPEAT({4 2},1,NUMINT/2-1)||{4 1})`;

DO UNTIL(EPOWER>POWER);

N1=N1+1;N2=NR#N1;

DF=N1+N2-2;

TCRIT=TINV(1-ALPHA,DF);

NFAC=1/N1+1/N2;

VAR=SIGSQ#NFAC;STD=SQRT(VAR);

CU=(DF#DEL##2)/(VAR#(TCRIT##2));

INT=CU-CL;INTL=INT/NUMINT;

CVEC=CL+(INTL#(0:NUMINT))`;

WCPDF=(INTL/3)#COEVECC#PDF('CHISQ',CVEC,DF);

ST=SQRT(CVEC/DF)#TCRIT;

EPOWER=WCPDF`*(CDF('NORMAL',(DEL-MUD)/STD-ST)-CDF('NORMAL',(-DEL-MUD)/STD+ST));

END;

PRINT ALPHA DEL MUD SIGMA SIGSQ NR POWER;

PRINT N1 N2 EPOWER[FORMAT=8.4];

QUIT;

Program B3

SAS IML program for calculating the optimal sample size *N*_2_ when the sample size *N*_2_ is fixed

PROC IML;

*USER SPECIFICATION PORTION;

*SAMPLE SIZE N2;N2=210;

*TYPE I ERROR & NOMINAL POWER;ALPHA=0.05;POWER=0.8;

*EQUIVALENCE BOUND;DEL=5.92;

*MEAN DIFFERENCE;MUD=2.2;

*STANDARD DEVIATIONS;SIGMA=9.78;

*END OF SPECIFICATION PORTION;

SIGSQ=SIGMA##2;NUMINT=1000;LC=NUMINT+1;CL=1E-10;

COEVECC=({1}||REPEAT({4 2},1,NUMINT/2-1)||{4 1})`;N1=3;

DO UNTIL(EPOWER>POWER);

N1=N1+1;

DF=N1+N2-2;

TCRIT=TINV(1-ALPHA,DF);

NFAC=1/N1+1/N2;

VAR=SIGSQ#NFAC;STD=SQRT(VAR);

CU=(DF#DEL##2)/(VAR#(TCRIT##2));

INT=CU-CL;INTL=INT/NUMINT;

CVEC=CL+(INTL#(0:NUMINT))`;

WCPDF=(INTL/3)#COEVECC#PDF('CHISQ',CVEC,DF);

ST=SQRT(CVEC/DF)#TCRIT;

EPOWER=WCPDF`*(CDF('NORMAL',(DEL-MUD)/STD-ST)-CDF('NORMAL',(-DEL-MUD)/STD+ST));

END;

PRINT ALPHA DEL MUD SIGMA SIGSQ N2 POWER;

PRINT N1 N2 EPOWER[FORMAT=8.4];

QUIT;

Program B4

SAS IML program for calculating the optimal sample sizes {*N*_1_, *N*_2_} to attain maximum power performance for a fixed cost

PROC IML;

*USER SPECIFICATION PORTION;

*COST COEFFICIENTS;CF=0;C1=4;C2=1;

*TOTAL COST;TCOST=400;

*TYPE I ERROR;ALPHA=0.05;

*EQUIVALENCE BOUND;DEL=5.92;

*MEAN DIFFERENCE;MUD=2.2;

*STANDARD DEVIATIONS;SIGMA=9.78;

*END OF SPECIFICATION PORTION;

SIGSQ=SIGMA##2;

NUMINT=1000;

LC=NUMINT+1;

CL=1E-10;

COEVECC=({1}||REPEAT({4 2},1,NUMINT/2-1)||{4 1})`;

N1Z=FLOOR(TC#SQRT(C2)/(C1#SQRT(C2)+C2#SQRT(C1)));

N1MIN=N1Z-3;

N2Z=FLOOR(TC#SQRT(C1)/(C1#SQRT(C2)+C2#SQRT(C1)));

N2MIN=N2Z-3;

N1MAX=FLOOR((TC-C2#N2MIN)/C1);

LN=N1MAX-N1MIN+1;

N1VEC=(N1MIN:N1MAX);

N2VEC=FLOOR((TC-C1#N1VEC)/C2);

TCVEC=CF+C1#N1VEC+C2#N2VEC;

POWERVEC=J(1,LN,0);

DO J=1 TO LN;

N1=N1VEC[1,J];

N2=N2VEC[1,J];

DF=N1+N2-2;

TCRIT=TINV(1-ALPHA,DF);

NFAC=1/N1+1/N2;

VAR=SIGSQ#NFAC;

STD=SQRT(VAR);

CU=(DF#DEL##2)/(VAR#(TCRIT##2));

INT=CU-CL;INTL=INT/NUMINT;

CVEC=CL+(INTL#(0:NUMINT))`;

WCPDF=(INTL/3)#COEVECC#PDF('CHISQ',CVEC,DF);

ST=SQRT(CVEC/DF)#TCRIT;

EPOWER=WCPDF`*(CDF('NORMAL',(DEL-MUD)/STD-ST)-CDF('NORMAL',(-DEL-MUD)/STD+ST));

POWERVEC[1,J]=EPOWER;

END;

MAXI=POWERVEC[1,<:>];

MAXN1=N1VEC[1,MAXI];

MAXN2=N2VEC[1,MAXI];

TCMAX=TCVEC[1,MAXI];

PMAX=POWERVEC[1,MAXI];

N1=MAXN1;

N2=MAXN2;

EPOWER=PMAX;

TC=TCMAX;

PRINT ALPHA DEL MUD SIGMA SIGSQ;

PRINT CF C1 C2 TCOST;TC=TCOST-CF;

PRINT N1 N2 TC EPOWER[FORMAT=8.4];

QUIT;

Program B5

SAS IML program for calculating the optimal sample sizes {*N*_1_, *N*_2_} to meet a designated power level for the least cost

PROC IML;

*USER SPECIFICATION PORTION;

*COST COEFFICIENTS;CF=0;C1=4;C2=1;

*TYPE I ERROR;ALPHA=0.05;POWER=0.8;

*EQUIVALENCE BOUND;DEL=5.92;

*MEAN DIFFERENCE;MUD=2.2;

*STANDARD DEVIATIONS;SIGMA=9.78;

*END OF SPECIFICATION PORTION;

SIGSQ=SIGMA##2;

NUMINT=1000;

LC=NUMINT+1;

CL=1E-10;

COEVECC=({1}||REPEAT({4 2},1,NUMINT/2-1)||{4 1})`;

NRZ=SQRT(C1/C2);

ZA=PROBIT(1-ALPHA);

ZB1=PROBIT(POWER);

ZB2=PROBIT(1-(1-POWER)/2);

IF MUD=0 THEN ZB=ZB2;

ELSE ZB=ZB1;

K=(SIGMA#(ZA+ZB)/(DEL-ABS(MUD)))##2;

N1Z=CEIL((1+1/NRZ)#K);

N2Z=CEIL(N1Z#NRZ);

N1MIN=MAX(5,N1Z-2);

N1MAX=N1Z+10;

N1VEC=(N1MIN:N1MAX);

LN=NCOL(N1VEC);

N2VEC=J(1,LN,0);

POWERVEC=J(1,LN,0);

DO J=1 TO LN;

N1=N1VEC[1,J];

N2=FLOOR(1/(1/K-1/N1));

N2=MAX(N2,6);

LOOP=0;

DO UNTIL (EPOWER>POWER | LOOP>200);

N2=N2+1;

LOOP=LOOP+1;

DF=N1+N2-2;

TCRIT=TINV(1-ALPHA,DF);

NFAC=1/N1+1/N2;

VAR=SIGSQ#NFAC;

STD=SQRT(VAR);

CU=(DF#DEL##2)/(VAR#(TCRIT##2));

INT=CU-CL;

INTL=INT/NUMINT;

CVEC=CL+(INTL#(0:NUMINT))`;

WCPDF=(INTL/3)#COEVECC#PDF('CHISQ',CVEC,DF);

ST=SQRT(CVEC/DF)#TCRIT;

EPOWER=WCPDF`*(CDF('NORMAL',(DEL-MUD)/STD-ST)-CDF('NORMAL',(-DEL-MUD)/STD+ST));

END;

N2VEC[1,J]=N2;

POWERVEC[1,J]=EPOWER;

END;

TCVEC=CF+C1#N1VEC+C2#N2VEC;

TCMIN=TCVEC[1,><];

MINIVEC=LOC(TCVEC=TCMIN);

N1MINVEC=N1VEC[1,MINIVEC];

N2MINVEC=N2VEC[1,MINIVEC];

PMINVEC=POWERVEC[1,MINIVEC];

TCMINVEC=TCVEC[1,MINIVEC];

PMAXMIN=PMINVEC[1,<>];

MAXMINI=PMINVEC[1,<:>];

N1MAXMIN=N1MINVEC[1,MAXMINI];

N2MAXMIN=N2MINVEC[1,MAXMINI];

PMAXMIN=PMINVEC[1, MAXMINI];

N1=N1MAXMIN;

N2=N2MAXMIN;

EPOWER=PMAXMIN;

TC=TCMIN;

PRINT ALPHA POWER DEL MUD SIGMA SIGSQ;

PRINT CF C1 C2;

PRINT N1 N2 EPOWER[FORMAT=8.4] TC;

QUIT;
